# Nondipping Blood Pressure Pattern Is Associated with the Development of Myocardial Injury after Noncardiac Surgery: A Single-center Prospective Observational Study: Erratum

**DOI:** 10.1097/ao9.0000000000000035

**Published:** 2026-10-19

**Authors:** Tomoharu Ukiya, Yoshihiro Yamaji, Takahiro Muramatsu, Sumire Yokota, Shiroh Isono

In the Original Investigation article titled “Nondipping Blood Pressure Pattern Is Associated with the Development of Myocardial Injury after Noncardiac Surgery: A Single-center Prospective Observational Study,” the Editor’s Perspective, “What This Article Tells Us That Is New” text incorrectly referred to “20-h blood pressure monitoring.”^1^ The correct information is as follows:

The 24-h blood pressure monitoring is feasible in preoperatively admitted patients and serves as a useful tool for detecting a nondipping blood pressure pattern.

The online version and PDF of the article have been corrected.

DOI: 10.1097/ao9.0000000000000035
